# Supplementary material for: Element- and momentum-resolved electronic structure of the dilute magnetic semiconductor manganese doped gallium arsenide
Source: Nat Commun. 2018 Aug 17;9:3306. doi: 10.1038/s41467-018-05823-z (PMC6098022; doi:10.1038/s41467-018-05823-z)
Supplement: Supplementary file 1 — Supplementary Information [file 41467_2018_5823_MOESM1_ESM.pdf]

Supplementary Information

Element- and momentum- resolved electronic structure of the dilute magnetic semiconductor $Ga_{1-x}Mn_xAs$

Slavomír Nemšák et al.

Supplementary note 1

Paths in **k**-space as determined by free-electron final-state analysis

In Supplementary Figure 1, we show the paths in **k**-space derived by fitting free-electron final-state calculations to the main band profiles for the undoped and doped samples, as shown in Figs. 4(a),(b) of the main text.

Supplementary Figs. 1(a),(b) show that the wide angle of acceptance of the momentum-resolving detector yields a curve that passes through various parts of the Brillouin zone (BZ), really sampling a great deal of the reduced zone as integrated over the full curve.

Within the three panels of Supplementary Fig. 1, one can also see the separate curves for the doped and undoped samples, and for two photon energies at conservative extremes of our measurement range. Noteworthy is first that the two different samples are shifted from one another when viewed in 3D (Fig. 1(a)) or in the k_z-k_y plane (Fig. 1(b)). This is due largely to a slight difference in the tilt angle as mounted in the spectrometer. Also important is that the range of energies changes k_z very little on the scale of the BZ dimensions (less than 2% of the Γ-X-Γ, which points along sample normal), justifying our neglect of this variation in our theoretical analysis.

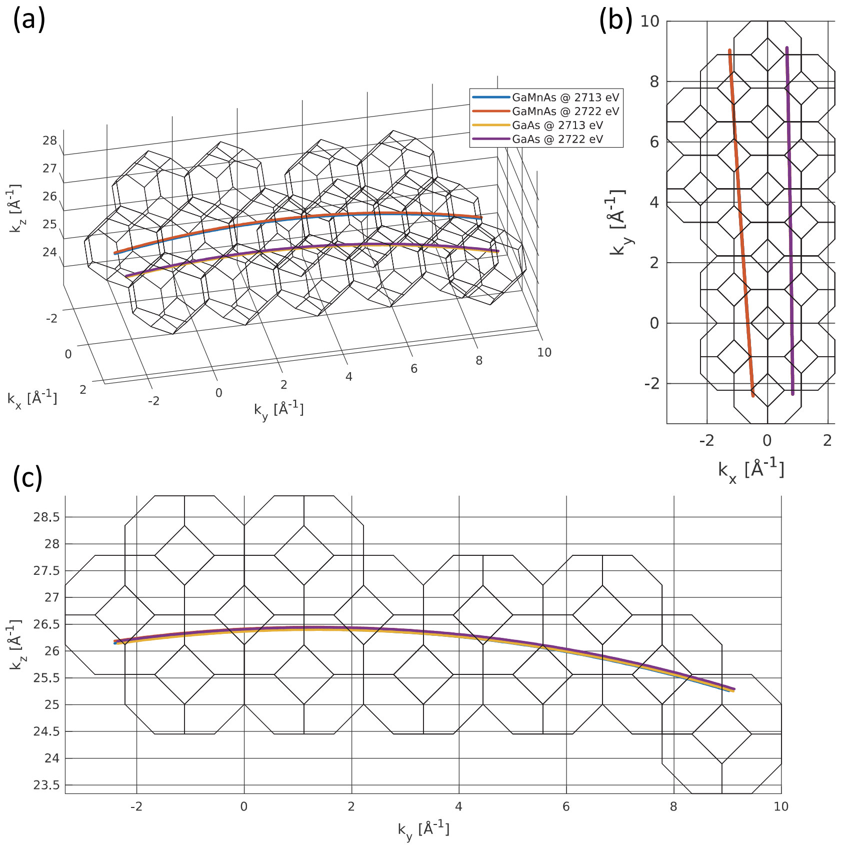

Supplementary Figure 1. Calculated trajectories in reciprocal space as measured along analyzer slits for undoped GaAs and doped Ga(Mn)As samples for two representative photon energies. Three different projections shown in panels (a)-(c).

Supplementary note 2

Alternate representation of the element- and **k**-resolved electronic structure

In Supplementary Figure 2, we show the equivalent of Figures 4(c), (d) in the main text, but with each of the elemental components shown separately, so as not to require the color scale. In some respects, this permits seeing more easily just where the different elemental contributions are found. It also makes it clearer that there is a Ga+Mn impurity band for (Ga,Mn)As at E_F for a detector angle of about 12° (roughly along sample normal), consistent with a strong feature there in the Bloch spectral functions of Figure 4(f).

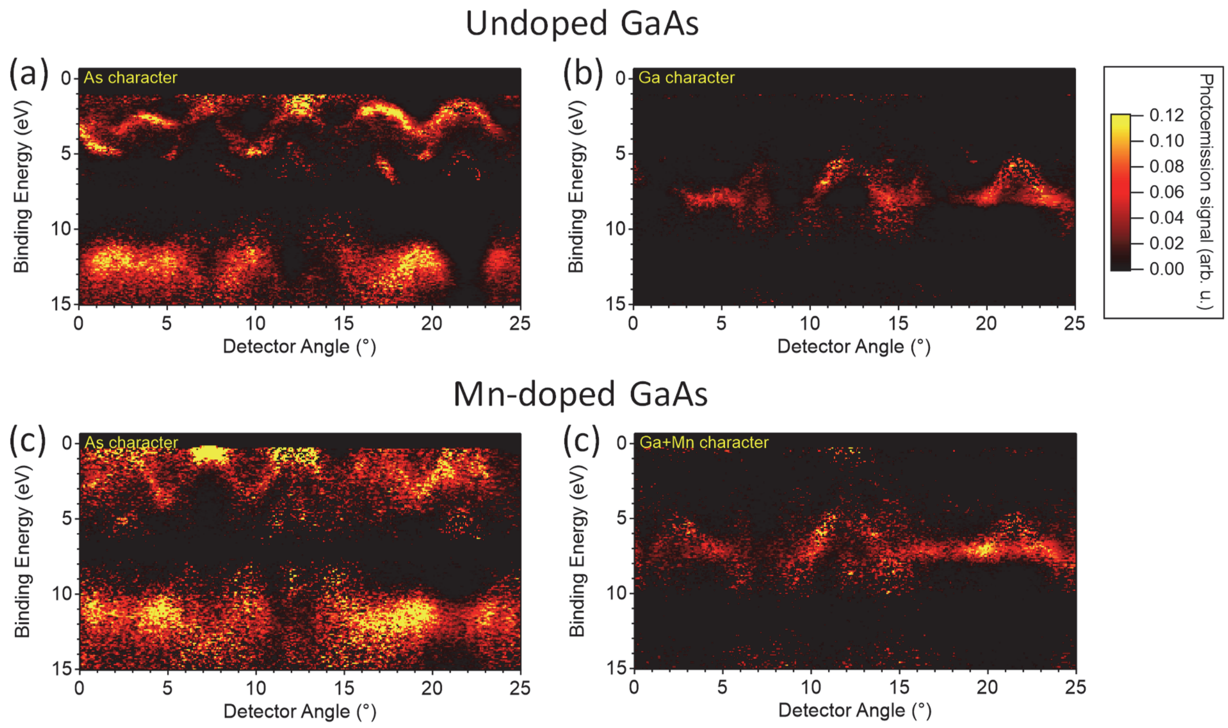

Supplementary Figure 2. Site-resolved electronic band structure. (a), (b) Maps shown for the undoped GaAs sample as deconvoluted by the SW experiment. (c), (d) Equivalent quantities extracted for the doped Ga(Mn)As sample.
